# Subject-Specific Surgical Planning for Hip Replacement: A Novel 2D Graphical Representation of 3D Hip Motion and Prosthetic Impingement Information

**DOI:** 10.1007/s10439-019-02260-x

**Published:** 2019-04-10

**Authors:** Arnab Palit, Richard King, Yolanda Gu, James Pierrepont, David Simpson, Mark A. Williams

**Affiliations:** 1 WMG, University of Warwick, Coventry, CV4 7AL UK; 2 Department of Trauma & Orthopaedics, University Hospitals Coventry and Warwickshire NHS Trust, Coventry, UK; 3 Optimized Ortho, 17 Bridge Street, Pymble, NSW 2073 Australia; 4 Corin Ltd, Corinium Centre, Cirencester, Gloucestershire GL7 1YJ UK

**Keywords:** Total hip replacement, Prosthetic impingement, Implant orientation, Hip joint, Activities of daily living

## Abstract

Prosthetic impingement (PI) following total hip arthroplasty (THA), which arises due to the undesirable relative motion of the implants, results in adverse outcomes. Predicting PI through 3D graphical representation is difficult to comprehend when all activities are combined for different implant positions. Therefore, the aim of the paper was to translate this 3D information into a 2D graphical representation for improved understanding of the patient’s hip motion. The method used planned implanted geometry, positioned onto native bone anatomy, and activity definitions as inputs to construct the 2D polar plot from 3D hip motion in four steps. Three case studies were performed to highlight its potential use in (a) combining different activities in a single plot, (b) visualising the effect of different cup positions and (c) pelvic tilt on PI. A clinical study with 20 ‘Non-Dislocators’ and 20 ‘Dislocators’ patients after 2 years of THA was performed to validate the method. The results supported the study hypothesis, in that the incidence of PI was always higher in the ‘Dislocators’ compared to the ‘Non-Dislocators’ group. The proposed 2D graphical representation could assist in subject-specific THA planning by visualising the effect of different activities, implant positions, pelvic tilt and related aspects on PI.

## Introduction

Total hip arthroplasty (THA) is a highly effective surgical intervention to relieve pain and restore function to patients with hip osteoarthritis.^3^,^26^ THA aims to enable the patients to return to their desired activities of daily living (ADLs) without restrictions and to maximise the life of the implants.^22^ If the biomechanical reconstruction is suboptimal, patients are at higher risk of poor function (e.g. limping, ongoing pain), complications (e.g. dislocation), and premature failure of the implants (e.g. implant loosening, excessive wear of the prosthetic joint surfaces). In such circumstances, the patient may have to undergo a revision THA, where the old implants are removed and new ones are inserted. Such revision operations are associated with significant complications (e.g. fractures, bone loss *etc*.), impose a significant cost burden on the healthcare provider, and are generally less reliable in terms of relieving symptoms and restoring function.^18^,^36^ Common reasons for revision surgery are aseptic loosening, wear, and recurrent dislocation.^1^,^7^,^15^,^16^,^20^,^32^,^33^ In these cases, there is often evidence of prosthetic impingement (PI) as the underlying biomechanical problem.^24^ PI occurs when the implanted femoral neck comes into contact with the rim of the acetabular cup during terminal motion of the hip. This type of contact collision can produce a tilting moment on the cup which may generate shear forces at the bone-implant interface, potentially contributing implant loosening.^8^,^21^,^33^,^37^ Further motion beyond the impingement point results in the femoral head being levered out of the acetabulum, such that it subluxes or dislocates.^7^,^11^,^15^,^27^ Besides, PI can also result in restricted range of movement, and increased pain.^2^,^25^ It is identified that PI is often the result of surgical misalignment of the femoral and acetabular components during THA.^12^,^17^ Therefore, patient-specific surgical planning, that can identify and visualise the effect of component position on PI, could reduce the chances of impingement, and therefore, improve post-surgical outcomes and minimise the need for revision surgery.

Historically, surgeons have planned hip replacements in two dimensions (2D), using conventional radiographs. Increasingly, however, these procedures are planned in three dimensions (3D) using a pre-operative CT scan. Such 3D plans can then be used for pre-operative analysis of the expected motion of the reconstructed hip, so that potential problems (such as PI) can be anticipated and mitigated by the surgeon. Recently, Schmid *et al.*^23^ developed a 3D computer-assisted platform which provided pre-operative THA planning using medical imaging and optical motion capture. Although the method showed a successful implementation, visualisation of different 3D hip motions and graphical representation of their effect on PI were limited. Previous efforts to visualise the dynamic 3D relationship between the components of a THA using 3D graphical representation have proved difficult to comprehend.^2^,^23^,^33^ Therefore, the aim of the paper was to develop a method which can translate 3D motion data and PI information into a 2D graphical representation for improved understanding of a patient’s expected hip motion following THA. Furthermore, a clinical study was performed with the following study hypothesis—number of patients with observed PI will always be higher in ‘Dislocators’ patients compared to ‘Non-Dislocators’ patients even for the basic hypothetical hip joint motion, and this could easily be visualised and comprehended through the developed 2D graphical representation. ‘Dislocators’ and ‘Non-Dislocators’ were two patient groups, which were categorised based on the incidences of hip joint dislocation after 2 years of THA. The developed 2D graphical plot from 3D hip joint motion information can potentially be used as a tool to explore the effect of different activities, component positions, pelvic tilt, and other factors on PI.

The paper is organised as follows. The conceptual novelty of translating 3D hip motion and PI information into a 2D graphical plot is described in the first part of materials and methods followed by its implementation. Thereafter, three case studies and one clinical study were included to highlight the various applications and validation of the method respectively. The rest of the paper describes the results followed by discussion.

## Materials and Methods

### Conceptual Novelty: 2D Graphical Representation of 3D Hip Motion and PI Information

**Figure 1:**
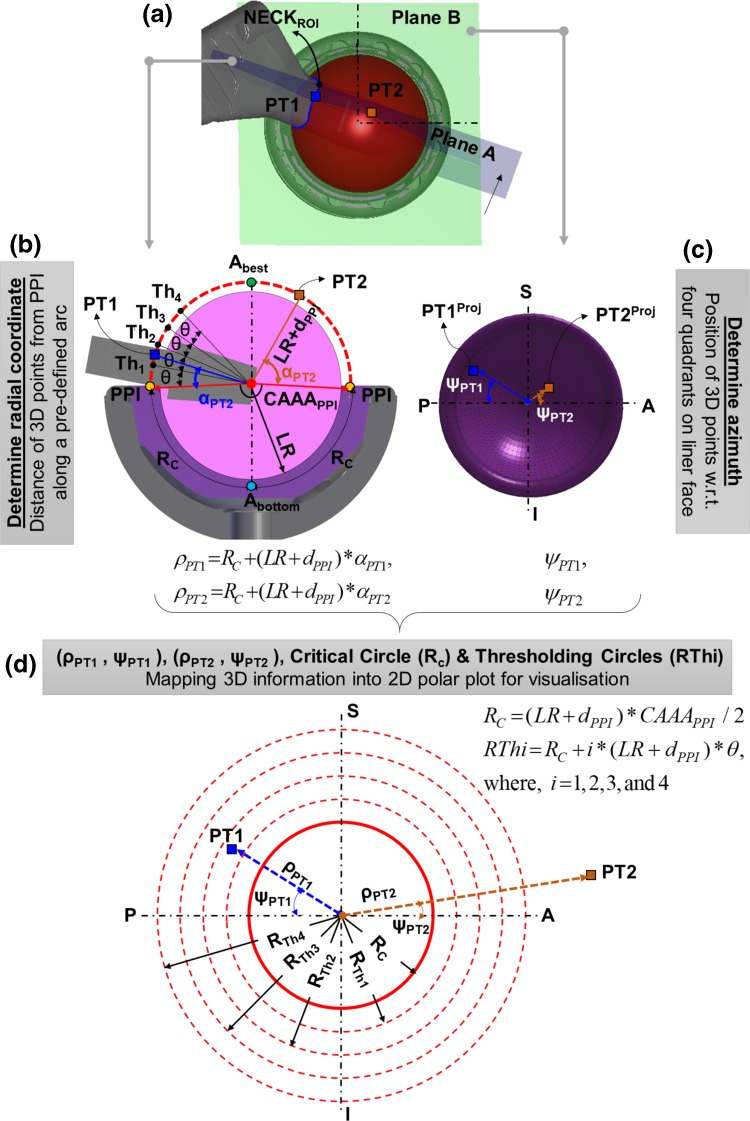
Conceptual novelty of the developed 2D graphical representation from 3D hip motion and PI information. (a) 3D representation of implants and NECK_ROI_-PT1 is a point on NECK_ROI_ whereas PT2 represents the location of PT1 after some time during a typical hip joint motion; (b) 2D cross sectional view of the implants on plane A (direction of view is shown by black arrow) to show the arc length for PT1 and PT2 which are to be mapped in 2D plot as radial coordinate; (c) 2D cross sectional view on plane B or LF plane (viewing from A_best_ towards A_bottom_) to show the azimuth for PT1 and PT2 which are to be mapped in 2D plot, S: Superior, I: Inferior, P: Posterior, A: Anterior; (d) Developed 2D polar plot from 3D hip motion information by mapping radial and azimuth of PT1 and PT2, critical and four threshold circles.

The main novelty of the work is the translation of 3D hip motion and PI information (Fig. 1a) into a 2D graphical representation though a 2D polar plot (Fig. 1d) without losing any information. This 2D visualisation is intended to provide easier understanding of the 3D information that is sometimes difficult to comprehend. In this novel method, radial coordinate $(\rho)$ represents how far the neck area would be from the rim of the liner during an activity (Fig. 1b), whereas azimuth $(\psi)$ depicts the 3D positions of the neck area into a 2D information (Fig. 1c).

### Determine the Radial Coordinate

The articulating surface between the femoral head and acetabular implant is located along the curved surface of the acetabular liner, radius of which is the liner radius (*LR*). The point of prosthetic impingement (PPI) (Fig. 1b, yellow dot) is defined as the first contact between the femoral neck and edge of the liner, where the liner face transitions to the rounded edge (fillet in CAD) of the articular margin. Some liners also use a flat chamfer rather than a fillet. Due to fillet or flat chamfer, the PPI is located at a small distance $(d_{PPI})$ away from the $LR$. This distance $(d_{PPI})$ depends on the fillet or flat champer at the edge of the articulating surface and on the geometry of the impinging prosthetic neck. Therefore, instead of considering entire geometry of the neck, only a specific region (NECK_ROI_) which could potentially contact the liner at PPI is considered for PI analysis. The NECK_ROI_ is defined by a cross-sectional boundary which is generated by cutting the neck geometry with a 3D hypothetical hemisphere of radius $(LR + d_{PPI} )$, and centre is at liner centre (Fig. 1a). In Fig. 1b, the red dotted half-circle (PPI → A_best_ → PPI) represents a 2D cross section of the 3D hypothetical hemispherical surface, and the NECK_ROI_ is the region where the red dotted curve intersects the neck. Therefore, any point on NECK_ROI_ will always move along the hypothetical hemisphere surface (Fig. 1a). When NECK_ROI_ movies towards A_best_ point (Fig. 1b), the possibility of PI reduces, and when it moves towards PPI, it increases. The Cup Articular Arc Angle $(CAAA)$ is most accurately measured by the points which are located just below (going from PPI towards A_bottom_ in Fig. 1b) the fillet or flat chamfered area of the liner.^35^ It could be thought that the fillet or chamfered area are removed by cutting it using a plane parallel to LF plane (plane B in Fig. 1a), and thereafter, the CAAA angle is measured by lines which are drawn perpendicular to the edge of the liner until they meet.^35^ It is a design feature of the liner which could be thought as the portion of a hemisphere of the acetabular bearing surface that articulates with the head of the implants.^34^ The fillet or chamfered area of the liner are ignored while measuring CAAA as it does not provide any support to the head. When considering PI, the equivalent angle is measured to the PPI which is located on the face of the liner (Fig. 1b), and is therefore marginally larger $\,(CAAA + \gamma)$. The value of $\gamma$ is calculated for the planned implant design. Therefore, the critical arc length (R_C_) (Fig. 1b) from A_bottom_ to PPI is defined by Eq. (1) which is the radius of the critical circle of the 2d polar plot (red continuous circle in Fig. 1d)

1 $$\begin{aligned}R_{C} = (LR + d_{PPI}) \times \frac{{CAAA_{PPI}}}{2}\;\;\\{\text{where,}}\,CAAA_{PPI} = CAAA + \gamma\end{aligned}$$

Suppose, PT1 is one of the points on NECK_ROI_ (blue square in Figs. 1a and 1b), and PT2, (orange square in Figs. 1a and 1b) represents the position of PT1 after some time steps of an activity. PT1 and PT2 make α_PT1_ and α_PT2_ angle respectively with the red arrow, defined by connecting liner centre and PPI. Therefore, the arc length in 3D (Fig. 1b), which defines the distance of the NECK_ROI_ from the rim of the liner (PPI) along the hypothetical hemisphere, is mapped to a radial distance in 2D plot (Fig. 1d) as defined by Eq. (2).

2 $$\begin{aligned} \rho_{PT1} = R_{C} + (LR + d_{PPI}) \times \alpha_{PT1} \hfill \\ \rho_{PT2} = R_{C} + (LR + d_{PPI}) \times \alpha_{PT2} \hfill \\ \end{aligned}$$

If during any time steps of an activity, *α*_*PT1*_ or *α*_*PT2*_ becomes zero (0) or negative, *ρ*_*PT1*_ or *ρ*_*PT2*_ will be equal or less than *R*_*C*_ respectively. This represents the situation of a PI which could easily be identified using the polar plot as part of NECK_ROI_ will be either touching or inside the critical red circle.

In order to provide a better visualisation of the relative distance of the NECK_ROI_ with respect to red critical circle in 2D polar plot, additional thresholding circles are included (red dotted circles in Fig. 1d). Four thresholding positions (Th1, Th2, Th3, and Th4) are defined on the 3D hypothetical hemisphere (Fig. 1b). When the NECK_ROI_ moves towards point Th1, Th2, Th3, and Th4 along the red circle of radius $(LR + d_{PPI} )$, the distance from the NECK_ROI_ to the rim of the liner increases, and therefore, the propensity of PI reduces, and vice versa. These thresholding points are separated with each other by an user defined angle $\theta$ (Fig. 1b) with an arc length $(LR + d_{PPI}) \times \theta$. Also, the first thresholding point (Th1) and PPI is also separated by same angle $\theta$. Therefore, the radius of the thresholding circles, which are related to thresholding points, are as follows

3 $$\begin{aligned}RThi = R_{C} + i \times (LR + d_{PPI}) \times \theta,\,\,\\{\text{where}}\,i = 1,\;2,\,3,\,{\text{and}}\,4\end{aligned}$$

The criteria of choosing thresholding points, and subsequently, thresholding circles depends on the value of angle $\theta$ which is entirely user defined. In this work, the angle $\theta$ is defined by some user-specific percentage of CAAA to make the thesholding points specific to implant design. However, value of angle $\theta$ could be chosen as a fixed value which would not depend on CAAA.

### Determine the Azimuth

The polar plot can be thought to be drawn on the plane of the liner face (LF) (plane B in Fig. 1a) which are divided into four anatomic quadrants (Fig. 1c) i.e. anterior–superior (S–A), posterior–superior (P–S), anterior–inferior (A–I), and posterior–inferior (P–I). These quadrants are created by constructing two perpendicular axes: Superior-Inferior and Posterior-Anterior which pass through the liner centre. The points of the NECK_ROI_, moving along the hypothetical 3D hemisphere surface, could be projected on the LF plane (Fig. 1c). Suppose, PT1 and PT2 are projected on LF plane to get the projected points PT1^Proj^ and PT2^Proj^ (Fig. 1c). These projected points create an angle *ψ*_PT1_ and *ψ*_PT2_ respectively with respect to Posterior-Anterior axis. These angles (*ψ*_PT1_ and *ψ*_PT2_) could then be used as the azimuth of the 2D polar plot (Fig. 1d).

### Implementation of the Method

**Figure 2:**
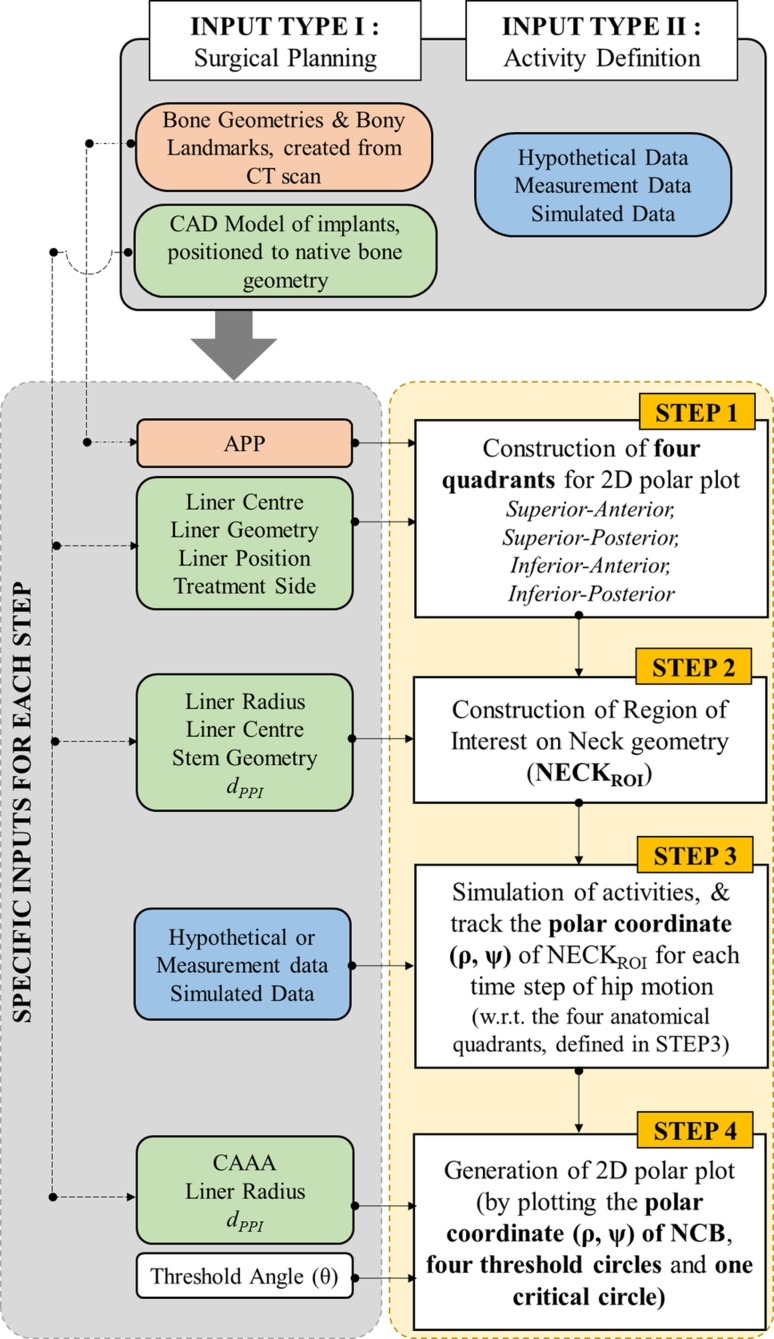
A brief overview of the method along with the inputs and steps involved to generate 2D polar plot form 3D hip motion and PI information for ADLs.

The following sections describe all the inputs and steps required for the method to translate 3D hip motion into a 2D graphical representation (Fig. 2).

### Inputs

There were two types of input required for the method—(A) Input Type I and (B) Input Type II (Fig. 2). (A) Input Type I was associated with 3D surgical plan, and dealt with the following main aspects: (a) CT scanning, (b) construction of bone geometries, (c) identification of bony landmarks, (d) CAD model of planned implants, and (e) planned implant positioning. Finally, bony landmarks and the implant geometries, positioned onto the native bone geometry according to the surgical plan, were used as Input Type I. (B) Input Type II represented the hip motion under consideration. This hip motion could be hypothetical activity (e.g. simple flexion, extension *etc*.), measured activity (e.g. using gait analysis), or simulated activity (e.g. generated using other software such as multi-body dynamics software).

### Step 1: Construction of Four Quadrants

**Figure 3:**
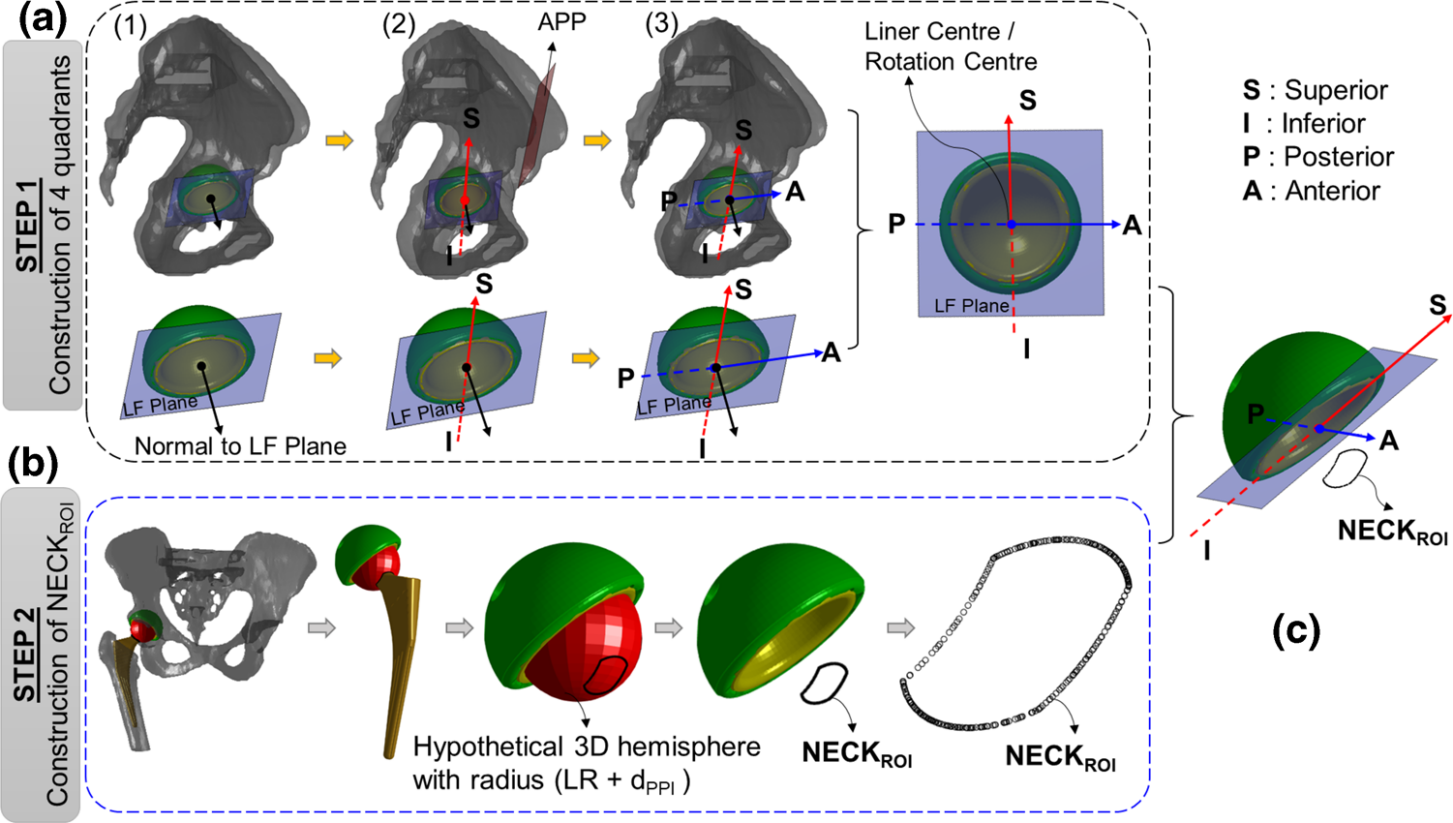
A graphical representation of step 1 and step 2. (a) Three step process to construct four quadrants of the proposed 2D plot; (b) construction of NECK_ROI_ from actual neck geometry; (c) a typical position of NECK_ROI_ with respect to four quadrants in 3D.

The four quadrants in the polar plot (Fig. 1d) are the four anatomic quadrants defined on LF plane (Fig. 3a (1)). Four inputs are required for this step (Fig. 2): (a) anterior pelvic plane (APP) which was defined by a best fit plane through left and right anterior superior iliac spines (ASIS) and the left and right pubic tubercles (PTUB), (b) liner centre, (c) liner geometry which was positioned onto the native bone geometry according to the surgical plan, and (d) treatment side (hip joint side). Firstly, a parallel plane of the APP was constructed which intersected the LF plane at liner centre (Fig. 3a (2)). The intersected line vector was the superior-inferior (SI) axis of the polar plot (Fig. 3a (2)). It divided the LF plane equally into two parts—posterior and anterior. Finally, posterior-anterior (PA) axis was defined by a line on LF plane, which was perpendicular to SI axis and passed through liner centre (Fig. 3a (3)).

### Step 2: Construction of NECK_ROI_

Only a specific region (NECK_ROI_), which would come first in contact with liner at PPI, was considered for PI analysis. The rest of the neck geometry was therefore ignored. Four inputs were required for this step (Fig. 2)—liner radius, liner centre, actual neck geometry, and *d*_*PPI*_. The NECK_ROI_ was then constructed by intersecting the neck/stem geometry with a hypothetical 3D hemisphere (Figs. 1a and 3b) of radius $\left({LR + d_{PPI}} \right)$ and centre at liner centre. This procedure was performed using Matlab function ‘fastMesh2Mesh’^29^ which employed Ray-Triangle intersection algorithm^13^ to automatically calculate the intersection points between two STL geometries with triangular mesh. It used triangular face ids and vertices of the hypothetical sphere and actual neck as inputs, and calculated a set of intersection points which was actually used as NECK_ROI_ (Fig. 3b). No assumption was made regarding the geometric profile of the neck. Therefore, this method would work for any neck geometry as the shape of the NECK_ROI_ will depict the true neck profile at a $\left({LR + d_{PPI}} \right)$ distance from liner centre. Figure 3c shows the 3D position of NECK_ROI_ with respect to four quadrants defined on LF plane during a particular time step of an activity.

### Step 3: Simulation of Activities to Get Polar Coordinate (*ρ,ψ*) of the NECK_ROI_ for Each Time Step

Input Type II (Fig. 2) was required to get the movement of NECK_ROI_. The radial coordinate $\left(\rho \right)$ was identified for each point on NECK_ROI_ using the following steps. (I) A vector $\left({v_{PL}} \right)$ was constructed using liner centre $(L_{0})$ and a point on NECK_ROI_$\left({P_{ROI}} \right)$ for a time step of the activity. The length of the vector ($v_{PL}$) was $(LR\, + \,d_{PPI})$ as all the points on NECK_ROI_ were located at $(LR\, + \,d_{PPI})$ distance from liner centre $(L_{0})$. (II) The angle $\left(\alpha \right)$ between the vector $v_{PL}$ and the LF plane signified that the point $P_{ROI}$ was $\alpha$ angle away from the LF plane. The liner is not a full hemisphere as the $CAAA_{PPI}$ is less than 180°. Therefore, the angle between LF plane and the axis (or plane), which was used to measured $CAAA_{PPI}$ (line/plane connecting liner centre and PPI (s), see Fig. 1b), is

4 $$\xi = \frac{{(\pi - CAAA_{PPI})}}{2}$$

Therefore, the point $P_{ROI}$ was effectively make an angle $(\alpha \, + \,\xi)$ with the line (or plane) connecting liner centre and PPI. (III) the arc length $\left({L_{arc}^{{P_{ROI}}}} \right)$ was then calculated as,

5 $$L_{acr}^{{P_{ROI}}} = (LR + \,d_{PPI}) \times (\alpha + \xi)$$

It represented that the point $P_{ROI}$ was $L_{arc}^{{P_{ROI}}}$ arc distance away from PPI. (IV) Finally, $L_{arc}^{{P_{ROI}}}$ was added to radius $R_{C}$ to get $\rho$

6 $$\rho = R_{C} + L_{arc}^{{P_{ROI}}}$$

If there is any impingement, $L_{arc}^{{P_{ROI}}}$ would be negative and $\rho$ would be less than $R_{C}$.

The azimuth of the same point $\left({P_{ROI}} \right)$ was identified using following steps. The projection $\left({P_{v}} \right)$ of the vector $v_{PL}$ on the LF plane was calculated, and thereafter, the angle ($\psi_{1}$) between $P_{v}$ and PA axis was measured. If the point $\left({P_{ROI}} \right)$ was located in quadrant Superior-Posterior or Superior-Anterior, the azimuth $\left(\psi \right)$ would be same as the calculated $\psi_{1}$. If the point $\left({P_{ROI}} \right)$ was situated on quadrant Inferior-Posterior or Inferior-Anterior, the azimuth $\left(\psi \right)$ would be $\left({2\pi - \psi_{1}} \right)$. Using this method, the polar coordinates $\left({\rho,\psi} \right)$ of all the points on NECK_ROI_ were calculated for each time steps (Fig. 2).

### Step 4: Generation of 2D Polar Plot

Finally, the polar coordinates $\left({\rho,\psi} \right)$ for all the points on NECK_ROI_ for each time step of an activity were plotted on the 2D polar plot along with the critical and four thresholding circles using four inputs—$CAAA_{PPI}$, liner radius, user defined thresholding angle $(\theta)$, and $d_{PPI}$ (Fig. 2). In this work, *θ* is defined as 5% of $CAAA_{PPI}$ for a suitable visualisation of the thresholding circles although any values of *θ* could be chosen as it is entirely user defined. If *θ* is very small, the thresholding circles would be very close together and it would look too cramped. On the other hand, large values of *θ* would locate the thresholding circles farther apart which would affect the visualisation.

## Case Studies

The 3D plans for a right THA were used to demonstrate the useful features of the 2D graphical representation in designing patient-specific THA planning. Three cases were considered: (a) Case I: The effect of moving the hip joint into different functional positions for a particular implant position; (b) Case II: The effect of changing acetabular cup orientation on PI; and (c) Case III: The effect of simulated pelvic tilt on PI for a particular implant position. Input Type I for the method (Fig. 2) was provided by experienced engineers at Corin Ltd, who provide a 3D THR planning service for surgeons. This retrospective analysis of the data from Corin was approved by Bellberry Human Research Ethics Committee (BHREC) - study number 2012-03-710. The position of acetabular components was defined by radiographic inclination and anteversion angle, as defined by Murray,^14^ and represented as inc/ant (e.g. 33°/25°) in this paper.

Case I (Table 1) was considered because it reflects the way in which surgeons assess the function of the THA during the surgery. After inserting the THA implants, it is normal practice for the surgeon to move the hip into relatively extreme functional positions to check that the hip is not prone to PI or dislocation. These positions typically include deep hip flexion with internal rotation and full extension with external rotation.^5^ Based on this common practice, four hypothetical hip movements were defined (Input Type II) for the Case I, and subsequently, the 3D hip motion and PI information for all these activities (Table 1) were included into the 2D polar plot.

**Table 1:** Definition of the hypothetical hip movements used for Case I.

Activities	Initial position	Final position
Extension (Extn)	Supine	10° Extension
External rotation at extension (ER_Ext_)	10° Extn, 0° ER_Ext_	20° ER_Ext_
Flexion (Flex)	Supine	90° Flexion
Internal rotation at flexion (IR_Flex_)	90° Flex, 0° IR_Flex_	35° IR_Flex_

These hypothetical activities are generally performed by surgeon during THA

Case II was explored because malorientation of the acetabular component during THA surgery is identified to increase the possibility of PI and dislocation. Three different acetabular component orientations were considered (Table 2) and the same four activities (Table 1) were simulated for each orientation. Polar plots were then generated to highlight the effect of changing acetabular orientation on predicted PI for these four activities.

**Table 2:** Description of Case II and Case III to highlight the effect of cup position and pelvic tilt respectively on PI through 2D polar plot.

Case II				Case III			
Cup position (inc/ant)	Activities	Pelvic tilt	2D Plot produced	Pelvic tilt	Activities	Cup position (inc/ant)	2D plot produced
33°/15°	All four	No	One	No pelvic tilt	Flex & IR	38°/25°	One
33°/25°	All four	No	One	Posterior pelvic tilt 5°	Flex & IR	38°/25°	One
43°/21°	All four	No	One	Anterior pelvic tilt 5°	Flex & IR	38°/25°	One

*inc/ant = radiographic inclination and anteversion angle, as defined in Murray 14

Case III was of interest because the pelvis is known to significantly flex (anterior tilt) and extend (posterior tilt) during ADLs, and therefore, an important factor during hip joint movement analysis. Three scenarios were considered: (a) no pelvic tilt, (b) posterior, and (c) anterior pelvic tilt^19^ (Table 3) for a given femur movement. It was assumed that pelvic tilt occurred maximally during flexion activity, and therefore, only the hip flexion and IR_Flex_ (Table 1) were analysed.

**Table 3:** Patient characteristics and intraoperative data.

Characteristic	Non-Dislocators (*n* = 20)	Dislocators (*n* = 20)
Sex (male/female)	13/7	9/11
Age (years)	65.1 ± 9.5	64.1 ± 8.43
Treatment side (left/right)	10/10	12/8
Cup size (diameter in mm)	53 (50–60)	51.8 (46–56)
Head size (diameter in mm)	34.2 (32–36)	34.6 (28–40)
Cup inclination (°)	40.3 ± 4.6	41.2 ± 8.1
Cup anteversion (°)	22.2 ± 6.9	25.1 ± 8.5
Stem anteversion (°)	10.4 ± 10.3	17.6 ± 7.6
$d_{PPI}$	1.1 ± 0.89	1.4 ± 1.1
$CAAA_{PPI}$	175.3 ± 4.9 (168°–180°)	175.8 ± 5.19 (163°–180°)

Typical representative cup positions within the safe zone^10^ were considered for case II and III to illustrate graphically the effect of different cup positions and pelvic tilt on PI respectively.

### Clinical Study and Validation of the Method

In order to validate the method, the data of patients who had previously undergone a THA was analysed. The anonymised data was provided by Corin Ltd, which was approved by BHREC (2012-03-710). Two groups of patients were studied, based on the outcome of their THA after 2 years: (a) ‘Non-Dislocators’ where there had been no postoperative episodes of hip dislocation; (b) ‘Dislocators’ where there had been at least one clinical episode of dislocation of the THA. Table 3 summarises the patient characteristics and intraoperative data used for this study. 3D models of the implants, positioned onto the native bone geometries, and bony landmarks were used as Input Type I which were extracted from post-operative CT scan by dedicated experienced engineers in Corin Ltd.

For Input Type II, two scenarios were considered as subject-specific hip motion data was unavailable for this study. In Scenario I, four hypothetical activities (Table 1) which are generally performed during surgery, were used. Three sets of extreme position of IR_Flex_ and ER_Ext_ were used in this study (Table 4) based on the values by Tannast *et al.*,^28^ whereas the final positions of flexion and extension were kept same (Table 4). In Scenario II, four pure joint motions at supine position were considered (Table 4). The extreme position of each activity was obtained from the reference value of Turley *et al.*^31^,^33^

**Table 4:** A summary of the definition of activities (Input Type II) used for Scenario I and II for the validation of the method through clinical study.

	Activities	Initial position	Final position
SCENARIO I: pure joint & combined hip motions			
SET1	Extension (Extn)	Supine	10° Extn
	Flexion (Flex)	Supine	90° Flex
	External rotation at extension (ER_Ext_)	10° Extn, 0° ER_Ext_	**20**° ER_Ext_
	Internal rotation at flexion (IR_Flex_)	90° Flex, 0° IR_Flex_	**25**° IR_Flex_
SET2	Extension (Extn)	Supine	10° Extn
	Flexion (Flex)	Supine	90° Flex
	External rotation at extension (ER_Ext_)	10° Extn, 0° ER_Ext_	**25**° ER_Ext_
	Internal rotation at flexion (IR_Flex_)	90° Flex, 0° IR_Flex_	**35**° IR_Flex_
SET3	Extension (Extn)	Supine	10° Extn
	Flexion (Flex)	Supine	90° Flex
	External rotation at extension (ER_Ext_)	10° Extn, 0° ER_Ext_	**30**° ER_Ext_
	Internal rotation at flexion (IR_Flex_)	90° Flex, 0° IR_Flex_	**45**° IR_Flex_
SCENARIO II: pure joint hip motion			
	Extension (Extn)	Supine	10° Extn
	Flexion (Flex)	Supine	90° Flex
	External rotation (ER)	Supine, 0° ER	45° ER
	Internal rotation (IR)	Supine, 0° IR	45° IR

It is well known that a significant proportion of THA dislocations occur due to PI.^7^,^11^,^15^,^24^ Therefore, the hypothesis of the study was that the number of patients with observed PI will be always higher in ‘Dislocators’ patients compared to ‘Non-Dislocators’ group even for the basic hypothetical activities considered through Scenario I and II with generalised range of motion (Table 4).

## Results

The results of three case studies are briefly descried below to highlight the various aspects of the 2D presentation of the 3D hip motion and PI information.

### Case Study I: Inclusion of Different Activities in a Same 2D Plot

**Figure 4:**
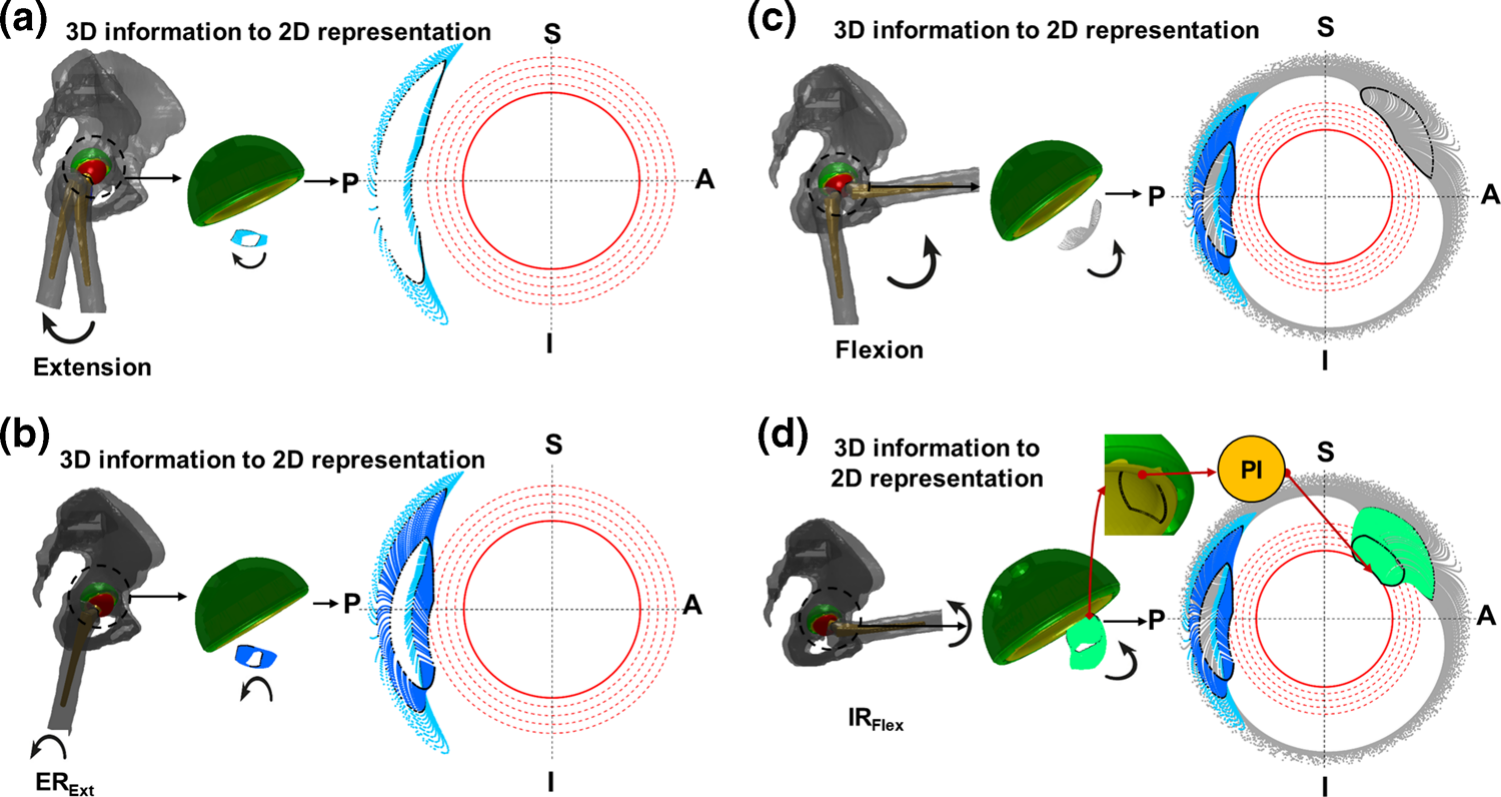
Results from ‘Case study I’ to show how a single 2D polar plot can be used to combine all the 3D information, generated from different activities. S, I, P, and A represent Superior, Inferior, Posterior, and Anterior respectively. (a), (b), (c) and (d) show the mapping of 3D NECK_ROI_ movement during extension (sky blue), EX_Ext_ (blue), flexion (grey), and IR_Flex_ (green) respectively into a 2D polar plot. The black NECK_ROI_ represents last time step of each activity.

Case study I displayed how 3D NECK_ROI_ movement during different activities could be combined in a single 2D polar plot (Fig. 4). 3D motion of NECK_ROI_ during extension activity was translated into 2D polar plot (Fig. 4a). It was observed that the chances of PI during extension activity was minimal as the NECK_ROI_ didn’t even cross the outermost threshold circle, and all of extension movement was confined around the posterior region of the liner (Fig. 4a). Similarly, the 3D NECK_ROI_ movement for both extension and ER_Ext_ were combined into 2D plot (Fig. 4b). It showed that the final position of ER_Ext_ crossed the outer most 4th threshold circle, and almost touched the 3rd threshold circle in the posterior region of the liner/cup, suggesting minimal propensity of PI (Fig. 4b). Similarly, it was observed that flexion of 90° had minimal possibility of PI as NECK_ROI_ only touched the third threshold circle in superior-anterior quadrant (Fig. 4c). However, the final position of the IR_Flex_ crossed the critical red circle in superior-anterior quadrant (Fig. 4d). This indicated that there was a high chance of PI due to 40° of IR_Flex_. The actual 3D impingement was shown using cup/liner geometry and NECK_ROI_ where it touched rim of the liner (Fig. 4d).

### Case Study II: Effect of Cup Orientation

**Figure 5:**
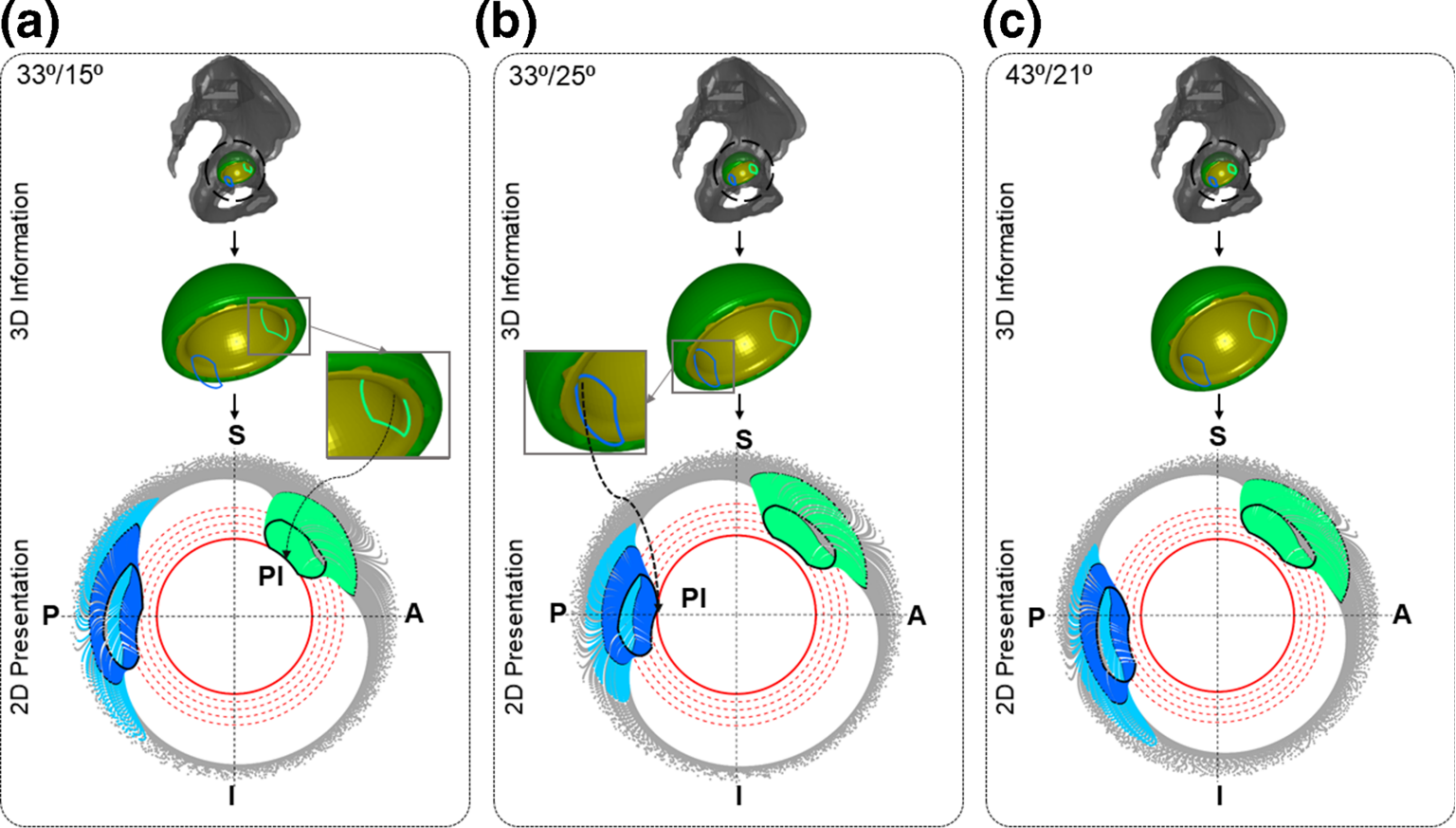
Results from Case Study II to show the effect of cup position on PI. (a), (b) and (c) show the 2D representation of 3D NECK_ROI_ movement during extension (sky blue), ER_Ext_ (blue), flexion (grey), and IR_Flex_ (green) activities for different cup positions at 33°/15°, 33°/25°, and 43°/25° respectively where first and second angles are inclination and anteversion respectively. S, I, P, and A represent Superior, Inferior, Posterior, and Anterior respectively. The black NECK_ROI_ in 2D polar plot represents last time step of each activity. The blue and green NECK_ROI_ in 3D geometry show the last time step of ER_Ext_ and IR_Flex_ respectively.

Case Study II showed how 2D polar plot can be used to highlight the effect of cup position on PI for same hip movement. It was observed that there was a chance of PI during IR_Flex_ at superior-anterior region of the liner when the acetabular cup was positioned with 33° inclination and 15° anteversion (Fig. 5a). The propensity of PI in ER_Ext_ was minimal as NECK_ROI_ only crossed 3rd threshold circle (Fig. 5a). On the other hand, there was a much higher possibility of PI in superior-posterior region during the ER_Ext_ when cup position changed to 33° inclination and 25° anteversion (Fig. 5b). However, the potential for anterior impingement during IR_Flex_ was now reduced as NECK_ROI_ was 1st threshold circle away. When cup position was changed to 43° inclination and 21° anteversion, the overall chances of PI was reduced, as the NECK_ROI_ was at least one threshold circle away from the acetabular margin for all simulated activities (Fig. 5c).

### Case Study III: Effect of Pelvic Tilt Orientation

**Figure 6:**
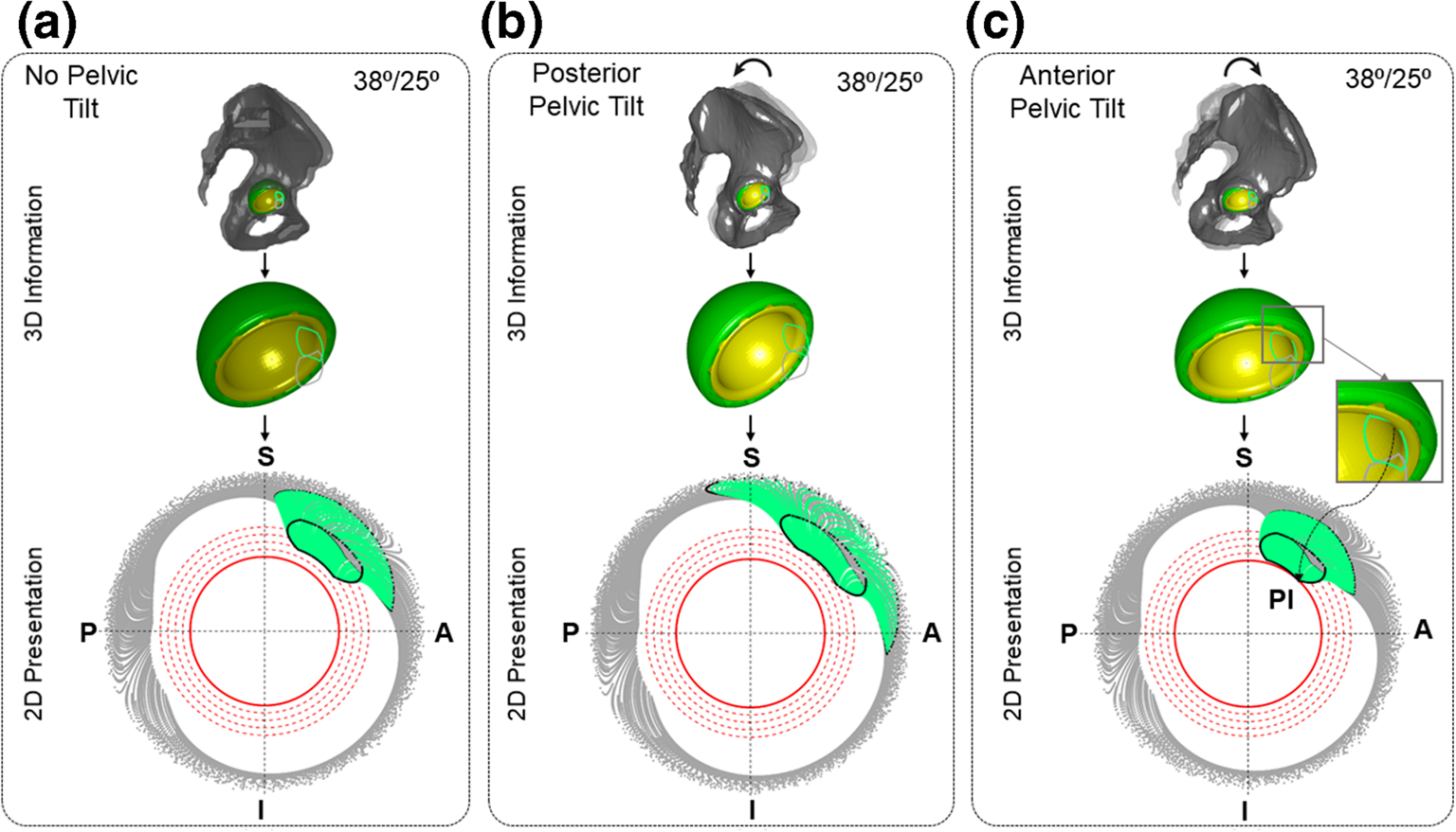
Results from Case Study III to show the effect of pelvic tilt on PI. (a), (b) and (c) show the 2D representation of 3D NECK_ROI_ movement during flexion (grey), and IR_Flex_ (green) activities when no-pelvic tilt, posterior and anetrior pelvic tilt are considered respectively for a particular cup position. S, I, P, and A represent Superior, Inferior, Posterior, and Anterior respectively. The black NECK_ROI_ in 2D polar plot represents last time step of each activity. The grey and green NECK_ROI_ in 3D geometry show the last time step of flexion and R_Flex_ respectively.

Case Study III depicted how 2D polar plot can be used to highlight the effect of pelvic tilt on PI propensity when performing the same pre-defined activities for a particular cup position (Fig. 6). NECK_ROI_ crosses 2nd threshold circles during last time step of IR_Flex_ when no-pelvic tilt was considered (Fig. 6a). If the subject had posterior pelvic tilt during flexion, it would further reduce the chance of PI, as NECK_ROI_ moved away from 1st to 3rd threshold circle (Fig. 6b). In contrast, anterior pelvic tilt clearly increased the possibility of PI, as the NECK_ROI_ touched the critical circle (Fig. 6c).

### Clinical Study and Validation of the Model

**Figure 7:**
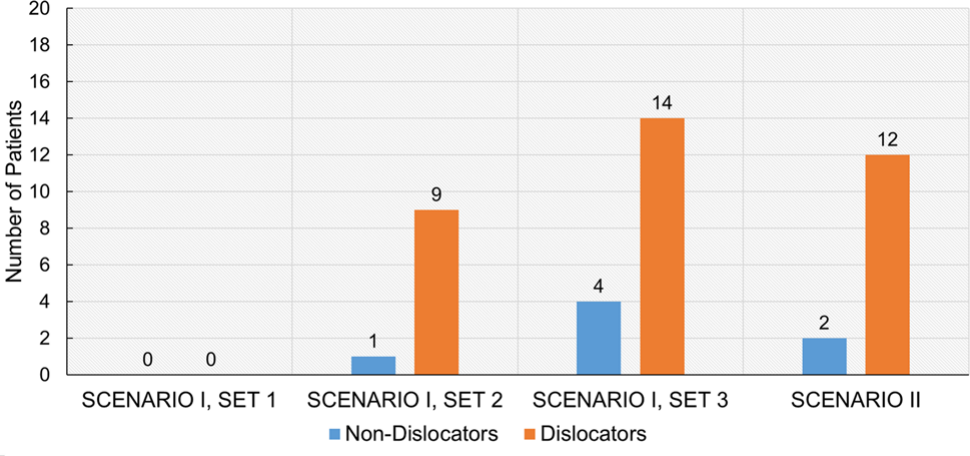
Results from clinical study which is used for validation of the method. Number of patients with observed PI is always higher for ‘Dislocators’ group compared to ‘Non-Dislocators’ group for each of the scenario.

**Figure 8:**
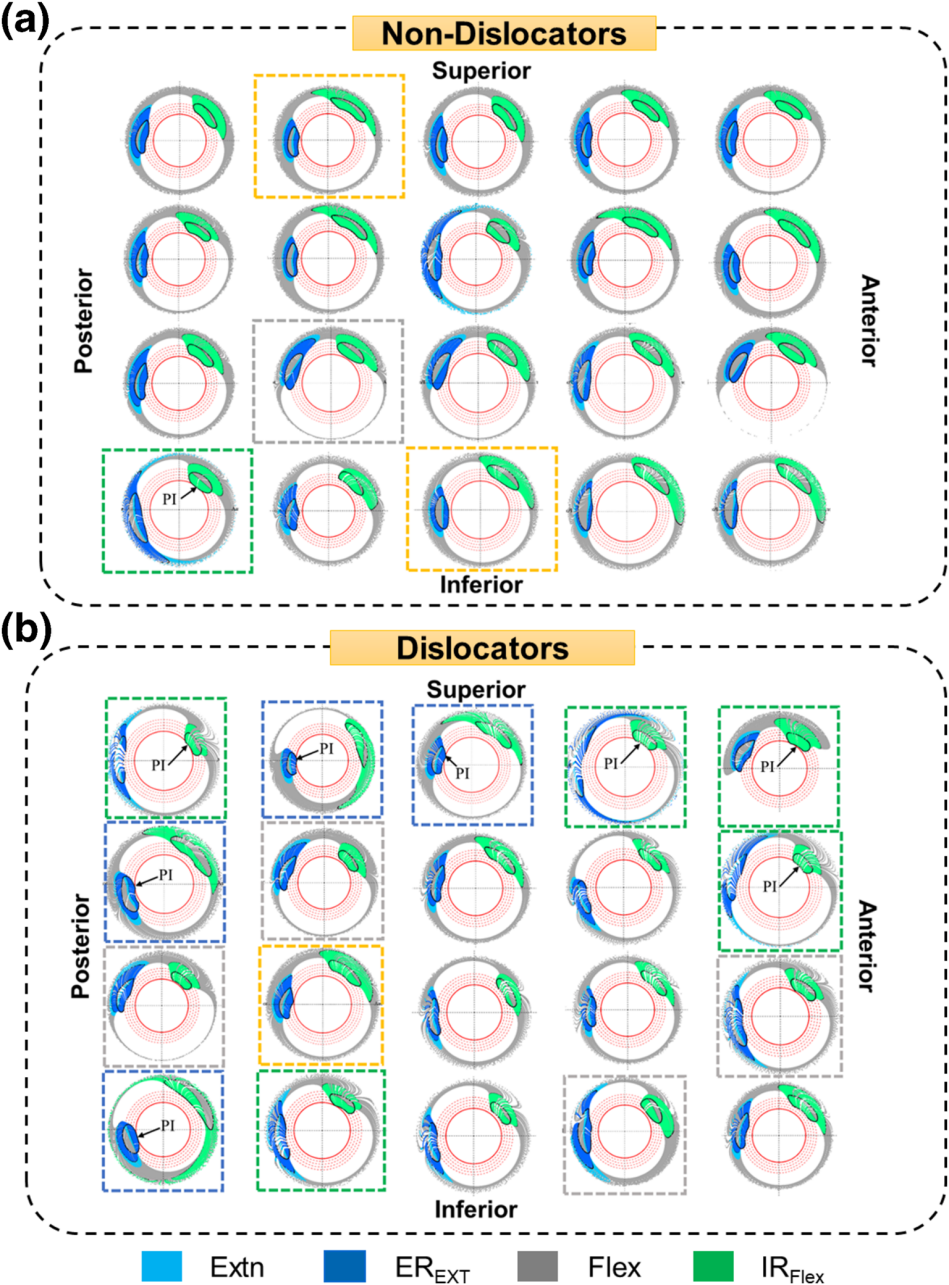
2D Graphical representation of the 3D hip motion considered in ‘Set 2’ of ‘Scenario-I’ for each (a) ‘Non-Dislocators’ and (b) ‘Dislocators’ patient. Each 3D hip motion is colour coded in 2D plot, and extreme position of each hip motion is highlighted with black colour. The green and blue dotted box show that the PI occur due to IR_Flex_ and ER_Ext_ respectively. The yellow and grey box show that those patient have high chance of PI due to ER_Ext_ and IR_Flex_ respectively if the extreme positions of these activities increase.

It was observed that the number of patients with PI was indeed always higher in the ‘Dislocators’ group compared to the ‘Non-Dislocators’ group (Fig. 7), which was consistent with the study hypothesis. For Set 1 in Scenario I, there was no detection (Fig. 7) as the ROM considered for ER_Ext_ and IR_Flex_ might not be extreme enough to cause any PI. When the ROM for these two activities increased, the number of detection was increased for both Set 2 and Set 3. However, the number of detections in ‘Dislocator’ was always higher-9 ‘Dislocators’ vs. 1 ‘Non-Dislocators’ in Set 2 and 14 ‘Dislocators’ vs. 4 ‘Non-Dislocators’ in Set 3. Scenario II depicted the similar results i.e. 12 ‘Dislocators’ patients with PI compared to 2 patients in ‘Non-Dislocators’ group. In Set 2, PI occurred due to IR_Flex_ and ER_Ext_ in 5 and 4 cases respectively (Fig. 8b), resulted in total 9 for ‘Dislocators’ group whereas IR_Flex_ caused only 1 case of PI in ‘Non-Dislocators’ (Fig. 8a). In Set 2, it was also observed that 4 and 1 patients in ‘Dislocators’ group had high chance of PI if the extreme position of IR_Flex_ and ER_Ext_ would increase respectively (Fig. 8b). This was evident from the results of Set 3 where final position of IR_Flex_ and ER_Ext_ were increased (Table 4). Similar observation was obtained when ‘Non-Dislocators’ group was considered. The patients with higher PI possibility (2 and 1 for IR_Flex_ and ER_Ext_ respectively) from Set 2 in ‘Non-Dislocators’ group (Fig. 8a) had PI when final positions of IR_Flex_ and ER_Ext_ were increased in Set 3 (Table 4).

## Discussion

This paper introduces a novel 2D graphical representation of 3D hip motion and PI information, which are difficult to visualise and comprehend. The proposed method has several features which could be modified according to the user’s requirements. Firstly, there were four threshold circles which were used to intuitively visualise the relative distance of the NECK_ROI_ from critical circle. Besides, the extreme position during any hip joint motion is subject-specific and varies considerably amongst patients. Therefore, when generalised extreme positions of an activity is used based on previous study, it was either over or under estimated for that particular patient. However, using threshold circles, it could be visually comprehended how much over or under estimation would affect PI (Fig. 8). Therefore, it entirely depends on the user whether to keep thresholding circles at all or how many thresholding circles should be used or what would be the radial separation distance between the thresholding circles. Secondly, non-linear scaling in radial direction of the 2D plot could be used to highlight further the positional differences of NECK_ROI_ for different scenarios. However, it should be noticed that the radius of the critical and four thresholding circles should also be changed using same non-linear scaling for consistency. In addition, type of non-linearity is also very crucial. Non-linearity functions such as exponential function which is monotonically strictly increases or decreases would be best. Thirdly, colour codes to represent critical and threshold circles, and locus of NECK_ROI_ for different activities are entirely user specific. Finally, it was never claimed that the values used in the paper for extreme positions of hip joint motion, cup positions or pelvic tilt were measured subject-specifically. These were typical representative values within their ranges as identified from the literature. These values were used just to illustrate various features of the proposed 2D plot, and therefore, the proposed method is not restricted to these values only. Therefore, the developed 2D plot could be a valuable tool to assist surgeons in the surgical planning of THA by analysing the effect of different ADLs, cup positions, pelvic tilt and other aspects on PI.

### Inclusion of Several Activities in a Single 2D Plot

One of the main advantages of the plot was that it could combine all of the 3D hip motion and PI information, generated during different activities, in a single 2D polar plot. As a result, it was easy to visualise which activity, and specifically, what range of movement of the activity, could cause PI. Recently, Hsu *et al.*^6^ developed a visualisation method based on similar concept i.e. mapping the movement of femoral neck with respect to the cup from a 3D sphere onto a 2D plane. However, Hsu *et al.*^6^ method tracked the movement of neck axis end point only whereas the proposed method considered the true shape of the actual neck geometry through NECK_ROI_. Therefore, effect of different stem geometries on PI, which might be difficult to highlight using Hsu *et al.*^6^ method, can still be visualised using the proposed method of the paper. Four hypothetical activities, which are quite commonly assessed by the surgeons during THA,^5^ were used in the case study to just demonstrate a useful feature of the plot. It was not claimed that the activities were subject specific, nor accurately measured. However, the extreme positions of the simulated activities were well within the ranges described in the literature.^9^,^28^,^30^,^31^ Different activities to those presented in the paper could also be visualised using different colours (Fig. 4). Also, the possibility of PI (if not explicitly observed) was recognised easily by comparing the position of NECK_ROI_ with respect to threshold circles. In addition, the location of impingement was presented in terms of anatomic quadrants i.e. anterior–superior, posterior–superior, anterior–inferior, and posterior–inferior. This information is likely to result in a more comprehensive assessment of the hip joint movement.

### Visualise the Effect of Cup Position on PI

The effect of cup position for a given set of activities could easily be visualised using the 2D polar plot (Fig. 5). It was observed that some cup positions created PI where other positions did not for a given femur movement (Fig. 5). In this study, three particular cup positions were analysed, but any combination of inclination/anteversion angles could be fed into the model to explore their effects on PI. Therefore, this plot can be used to intuitively suggest a better cup position for the patient provided they are compatible with other selection criteria. Cup positions, combined anteversion or implant geometries are associated with surgical plan, and therefore, these aspects are the part of ‘Input Type I’. As a result, the effect of these factors on PI could easily be visualised without any change in the steps (Step 1 to Step 4 in Fig. 2) of the proposed method.

### Visualise the Effect of Pelvic Tilt on PI

Pelvic rotations have a direct effect on the functional orientation of the cup/liner,^19^ and subsequently, on PI. Using Case III, it was demonstrated that the proposed 2D plots could visualise the effect of pelvic tilt orientation (posterior or anterior) on PI for a given femur movement and cup positions (Fig. 6). These orientations and the amount of pelvic tilt are subject-specific and also depend on type of activities. Therefore, some hypothetical but clinically relevant values of pelvis tilt^19^ were used for a given hypothetical femur movements as the objective of the Case III was only to highlight the capability of 2D plot in visualising the effect of pelvic tilt on PI. However, effect of any activity-specific pelvic tilt on PI for any subject can easily be visualised using the proposed 2D plot.

### Clinical Study and Validation of the Method

In this paper, the clinical study was used as a validation procedure of the proposed method rather than finding any clinical significance. For different scenarios, the proposed method always identified more patient with observed PI in ‘Dislocators’ group compared to ‘Non-Dislocators’ group, which corresponded to study hypothesis. It can therefore be inferred that the implementation of the proposed method was correct. In addition, the clinical study revealed that the method can easily be used for any implant geometries and their positions (cup inclination/anteversion or combined anteversion *etc*.) as highlighted in Table 1.

One of the advantages of this 2D graphical representation was that the surgeons could predict which activity might cause PI if the extreme position of the activity would increase a little (Fig. 8). For a particular activity, there might not be any observed PI in the 2D plot. However, the location of NECK_ROI_ with respect to the thresholding circles could convey the possibility of potential PI if the extreme position of the activity would increase. There is a strong trend to use 3D-data in routine clinical practice especially in Orthopedics. Availability of 3D-CT data might eventually help to represent PI analysis through digital display of 3D model which might reflect the actual realistic scenarios. However, according to the authors’ opinion, it would be difficult to visualise and comprehend the actual distance between the rim of the liner and the neck from the digital display of 3D model as this distance is not a linear distance, rather it is a nonlinear arc distance. It could be observed from Fig. 4 that the actual distance of the NECK_ROI_ from the rim of the cup was not comprehended well from the 3D representation although when this curvilinear distance was mapped to a linear distance in a 2D plot, it was easily comprehended and comparable. Another advantage of this 2D plot is that it is easier to understand in printing and book format compared to the 3D model. During primary and revision surgery, the surgeon has to decide whether to change the orientation of the acetabular component, the femoral component, or both. There is currently limited functional information available to the surgeon planning primary and revision surgery that can inform the specific changes to the THA that are necessary, and therefore, this method has the potential to aid pre-operative surgical decision making in these challenging surgical cases.

## Limitations

Firstly, the hypothetical activities and the pelvic tilt, used in the case studies, were defined based on the generalised range of motion data from the literature as subject-specific data was not available. If accurate direct measurement was available, this could easily be fed into the model to generate more accurate results. Secondly, there was no direct validation of the method. It is not possible at present to accurately directly record pelvic tilt, femoral movements, and the presence of impingement simultaneously in real-time in patients. Our indirect validation, performed through the clinical study, produced results which are consistent with clinical experience. Thirdly, the effects of changing the design of the femoral component or combined ante(version) on PI were not explicitly analysed in this paper, but the method would readily permit this as this information is only related to Input Type I. Finally, it must be acknowledged that there are other biomechanical issues such as bone impingement^4^ that can compromise the function and longevity of the THA which this method does not consider.

## Final Remarks

This paper introduces a novel concept of translating 3D hip motion and PI information into a 2D graphical representation through a 2D polar plot. 3D hip motion information of several activities could be combined into this single 2D plot to identify the activities which are prone to cause PI, and the anatomic region of the cup where this impingement would occur. In addition, this 2D plot is easier to comprehend, and therefore, could potentially be used as a tool for exploring the effect of different cup positions, pelvic tilt, combined anteversion and many other aspects of the surgical procedure on PI propensity to inform patient-specific primary and revision THA planning.
